# Sensory-Motor Interactions for Vocal Pitch Monitoring in Non-Primary Human Auditory Cortex

**DOI:** 10.1371/journal.pone.0060783

**Published:** 2013-04-08

**Authors:** Jeremy D. W. Greenlee, Roozbeh Behroozmand, Charles R. Larson, Adam W. Jackson, Fangxiang Chen, Daniel R. Hansen, Hiroyuki Oya, Hiroto Kawasaki, Matthew A. Howard

**Affiliations:** 1 Human Brain Research Lab, Department of Neurosurgery, University of Iowa, Iowa City, Iowa, United States of America; 2 Speech Physiology Lab, Department of Communication Sciences and Disorders, Northwestern University, Evanston, Illinois, United States of America; University of Texas Medical School at Houston, United States of America

## Abstract

The neural mechanisms underlying processing of auditory feedback during self-vocalization are poorly understood. One technique used to study the role of auditory feedback involves shifting the pitch of the feedback that a speaker receives, known as pitch-shifted feedback. We utilized a pitch shift self-vocalization and playback paradigm to investigate the underlying neural mechanisms of audio-vocal interaction. High-resolution electrocorticography (ECoG) signals were recorded directly from auditory cortex of 10 human subjects while they vocalized and received brief downward (−100 cents) pitch perturbations in their voice auditory feedback (speaking task). ECoG was also recorded when subjects passively listened to playback of their own pitch-shifted vocalizations. Feedback pitch perturbations elicited average evoked potential (AEP) and event-related band power (ERBP) responses, primarily in the high gamma (70–150 Hz) range, in focal areas of non-primary auditory cortex on superior temporal gyrus (STG). The AEPs and high gamma responses were both modulated by speaking compared with playback in a subset of STG contacts. From these contacts, a majority showed significant enhancement of high gamma power and AEP responses during speaking while the remaining contacts showed attenuated response amplitudes. The speaking-induced enhancement effect suggests that engaging the vocal motor system can modulate auditory cortical processing of self-produced sounds in such a way as to increase neural sensitivity for feedback pitch error detection. It is likely that mechanisms such as efference copies may be involved in this process, and modulation of AEP and high gamma responses imply that such modulatory effects may affect different cortical generators within distinctive functional networks that drive voice production and control.

## Introduction

In order to maintain effective vocal communication, the brain needs to finely control the acoustical parameters of self-generated vocal sounds. This important function requires online monitoring and control of the vocal motor commands that drive voice production via sensory feedback mechanisms. A well-accepted theory [1] has proposed that the brain detects and corrects for motor errors through comparing the actual sensory feedback to an internal representation of predicted feedback that is possibly provided by motor-related mechanisms such as efference copies [2]–[4]. Behavioral studies have supported this theory in the audio-vocal system by showing that applying an external perturbation to voice auditory feedback activates brain mechanisms that compensate for vocal errors in a reflexive manner [5]–[9].

Electrophysiological recordings in animals [10]–[12] and humans [13]–[19] have shown that the auditory cortical responses to normal (unaltered) voice feedback are attenuated during active vocal production compared with passive listening to the playback of self-produced voice. This effect is suggested to be associated with motor-driven mechanisms (e.g. efference copies) that suppress neural responses to predictable sensory feedback input. It has also been hypothesized that the brain may utilize efference copies to distinguish self-produced voices from those generated by an external source (e.g. other speakers) [17], [20]. In addition, when a pitch perturbation was applied to voice auditory feedback, some auditory cortical responses were found to be enhanced during active vocalization compared with passive listening to playback [21], [22]. These findings suggest that efference projections originating from motor-related areas may change tuning properties of auditory cortical neurons in such a way as to increase their sensitivity for accurate detection and correction of unexpected feedback changes (errors) during vocal production.

In the present study, we investigated the neural mechanisms of audio-vocal integration for voice pitch monitoring by recording directly from auditory cortex of human subjects using high-resolution electrocorticography (ECoG) signals in an auditory feedback pitch perturbation paradigm. We hypothesized that active vocal production (speaking) would lead to the enhancement of the evoked potentials in response to pitch perturbation in cortical auditory areas. In addition, due to previously reported correlation between high gamma oscillations (70–150 Hz) and single-unit and BOLD activity [23]–[26], we predicted that speaking would elicit stronger high gamma band power responses to feedback pitch perturbations compared with the playback condition. Furthermore, we predicted that the response changes observed in auditory cortex would be limited to discrete portions of the superior temporal gyrus (STG), given what is known about the response properties and connectivity of human STG.

## Materials and Methods

### Subjects

Ten subjects (8 males, 2 females; mean age 34.4 years (range 20–48); all right-handed except for 1 female (R180)) undergoing surgical treatment of medically intractable epilepsy volunteered to participate in this research protocol. Written informed consent was obtained from every subject and all research protocols were approved by the University of Iowa Human Subjects Review Board. Subjects did not incur any additional medical risks by participating in these studies.

All subjects completed an extensive pre-surgical assessment including detailed neurological examination, brain imaging (MRI, PET, and SPECT), and neuropsychological evaluation that confirmed normal speech and language functions (Greenlee et al., 2011). No anatomic lesions were observed in the cortical regions of interest to this study (e.g. posterior inferior frontal gyrus, lateral peri-Rolandic cortex, STG) in any subject. Audiometric testing was conducted and all patients were found to have normal hearing. All subjects underwent preoperative sodium amobarbital (Wada) testing to determine hemispheric language dominance. The left hemisphere was dominant for language in nine subjects and bilateral language representation was noted in one subject (L162). Experiments were conducted in a specially designed and electromagnetically-shielded private patient suite in the University of Iowa General Clinical Research Unit. All subjects participated in a multi-disciplinary epilepsy surgery evaluation and treatment protocol. Each subject was deemed an appropriate candidate for surgical placement of intracranial recording arrays for the purpose of recording and anatomically localizing seizure events in order to then excise the seizure focus.

### Electrode Implantation

Custom manufactured high-density electrode arrays (see below) were implanted on the pial surface of the exposed brain regions. The surface recording arrays consisted of 96 platinum-iridium disc electrodes embedded within a silicon sheet with 5 mm center-to-center spacing and 3 mm contact diameter (Ad-Tech, Racine, WI). The exact position of the recording grid differed somewhat between subjects as grid placement was based on patient-specific clinical considerations for each subject. In all subjects the coverage provided by the array included significant portions of the STG, including a previously described posterior lateral superior temporal auditory area (PLST [27]. The electrodes remained in place during a 14-day hospital stay during which time the patients underwent continuous video-EEG monitoring. This high-resolution EEG monitoring confirmed that the peri-Sylvian cortical areas pertinent to this study did not show abnormal inter-ictal activity. At the completion of the monitoring period, the electrodes were removed and the seizure focus was resected. Resections in all cases were restricted to the anterior temporal and mesial temporal lobe structures. The resections did not involve the STG. Separate electrodes were implanted in the subgaleal space over the vertex to serve as reference contacts.

### Electrode Localization

The exact position of each recording electrode was localized using a combination of high-resolution digital photographs taken intra-operatively during electrode placement and removal, as well as thin-cut pre- and post-implantation MR (0.78×0.78×1.0 mm voxel size) and CT (0.45×0.45×1.0 mm voxel size) scans. Pre- and post- implantation CT and MRIs were co-registered using a 3-D rigid-fusion algorithm implemented in FMRIB’s Linear Image Registration Tool [28]. Coordinates for each electrode obtained from post-implantation MRI volumes were transferred to pre-implantation MRI volumes. The location of every contact relative to visible surrounding brain structures was compared in both pre- and post-implantation MRI volumes. Such comparisons are useful since implantation of surface electrodes displaces the cerebral hemisphere medially with superficial brain tissue being distorted more than deeper structures. The resultant electrode locations were then mapped to a 3-D surface rendering of the lateral cerebral convexity. The estimated overall error in electrode localization using these techniques does not exceed 2 mm.

### Experimental Design

The experiment consisted of two blocks of speaking and two blocks of listening to playback. During the speaking blocks, subjects were asked to actively produce and sustain a steady vocalization of the vowel sound/a/for approximately 2 seconds at their conversational pitch and loudness. This vocal task was repeated 40 times during each block with subjects taking short breaks (1–2 seconds) between successive utterances, and vocalizing at their own pace. During each vocalization trial, subjects were presented with a downward (−100 cents) pitch shift stimulus in their voice auditory feedback. The onset of pitch shift stimuli in each vocalization trial was randomized between 500–1000 ms after the vocalization onset and the total duration of stimuli was 200 ms. These parameters have been well-studied in normal subjects using non-invasive techniques [21]. Each speaking block was recorded and immediately played back to the subject such that each subject passively listened to the pitch shift stimuli (PSS) in the auditory feedback of their own recorded vocalizations. The total duration of each block was approximately 5–8 minutes and the subjects were given short breaks (∼2 minutes) between successive blocks.

### Instrumentation

Each subjects’ voice was recorded using a condenser microphone (Beta 87C, Shure, Niles, IL), amplified (Ultralite MK3, MOTU, Cambridge, MA) and pitch-shifted through a harmonizer (Eclipse, Eventide, Little Ferry, NJ). All parameters of the pitch-shift stimuli (onset time, duration, magnitude etc.) were controlled by MIDI software (Max/MSP v5.0, Cycling ’74, San Francisco, CA) running on a standard laboratory computer. The Max/Msp software generated a TTL pulse to mark the onset of pitch-shift stimuli for time-locked averaging of the recorded brain potentials. The voice, feedback and TTL signals were recorded on a TDT data acquisition system (System3, Tucker Davis Technologies, Alachua, FL) with sampling frequency of 12 kHz. Subjects received their voice auditory feedback through a pair of earphones (ER4, Etymotic, Elk Grove, IL) placed in custom-fit, vented insert ear molds. A 10 dB gain was adjusted between the voice and its auditory feedback to partially mask the effect of air-borne and bone-conducted feedback. At conversational levels, subjects maintained their voice loudness at about 70–75 dB and received their feedback (through earphones) at 80–85 dB during speaking blocks. The feedback loudness during speaking was measured using a sound level meter implemented in the Max/Msp program and the feedback gain was adjusted to ensure that the feedback loudness during playback was the same as that during speaking blocks. We acknowledge that it is not possible to fully equalize the total sound energy delivered to the ear between speaking and playback blocks as bone conduction cannot be eliminated during speaking.

### Electrophysiological Recording

Research recordings were initiated several days post-implantation after subjects had fully recovered from implantation surgery. The electrocorticogram (ECoG) signals were recorded directly from the cortical surface of awake patients during the presentation of pitch-shifts in their voice auditory feedback. ECoG recordings were simultaneously acquired with voice, feedback and TTL signals using the TDT system under both speaking and playback blocks. The ECoG signals were first filtered (1.6–1000 Hz anti-aliasing filter) and then digitized with a sampling frequency of 2034.5 Hz. Digitized data were then resampled offline at 2000 Hz in MATLAB software (Mathworks, Natick, MA) for further processing.

### Data Analysis

**Figure 1:**
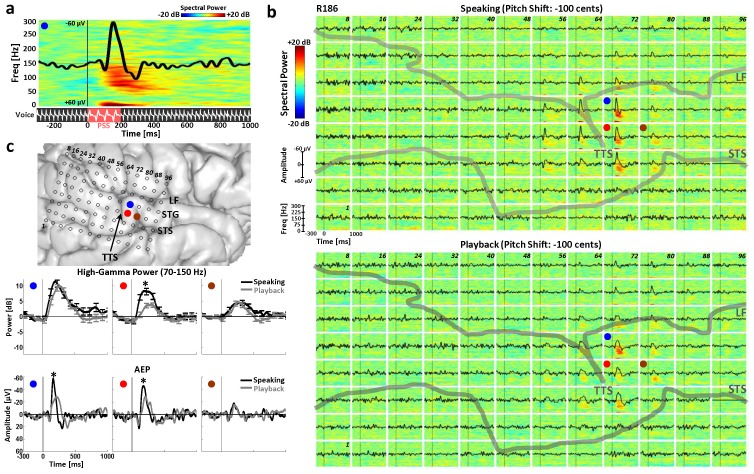
Exemplar STG Response Pattern to PSS. **a)** Time-frequency plot of spectral power (0–300 Hz) of the ECoG signal along with the overlaid AEP responses (solid black trace) to −100 cents pitch shift stimulus (PSS; red shading) in the auditory feedback during speaking of the vowel sound “a” for a single contact on the STG in a right-hemisphere implanted subject (R186). The solid vertical line marks the onset of the stimulus (0 ms) and the epoch window ranges from −300 to 1000 ms from the PSS onset. Subject’s voice and its pitch-shifted feedback are schematically shown in the lower panel. **b)** Time-frequency plots of spectral power along with overlaid AEPs for all 96 contacts of the right-hemisphere temporal lobe grid during speaking (top panel) and playback (bottom panel) conditions in subject R186. The thick gray lines show the lateral fissure (LF) and superior temporal sulcus (STS) and the gyrus in between them is the superior temporal gyrus (STG). The transverse temporal sulcus (TTS) is also labeled in each plot. **c)** Anatomical location of the implanted right-hemisphere temporal lobe grid with LF, STS, STG and TTS marked. The blue, red and brown circles mark three example contacts on the STG in vicinity of the TTS for which the high gamma (70–150 Hz) and AEP responses are separately shown on the bottom with overlaid traces during speaking and playback conditions. The error bars in the high gamma plots are the standard error from the mean (SEM) and the “*” marks show significant modulations during speaking compared with playback.

Recordings from all 96 ECoG channels were manually inspected to ensure that they were not contaminated by epileptiform activity or artifact and electrical line noise was removed using a notch filter at 60 Hz. Average evoked-potentials (AEPs) were calculated by first band-pass filtering (0.5–20 Hz, −24 dB/Oct) the ECoG signals and then segmenting them into epochs encompassing −300 ms before to 1000 ms after the onset of each pitch-shift stimulus, as marked by the TTL pulses. The time stamps of stimuli were further verified by extracting the pitch contour of the voice feedback channel and confirming that the pitch-shift onset lined up with the onset of the TTLs. Following segmentation, artifact rejection was carried out by excluding ECoG epochs with amplitudes exceeding +/−500 µV. Individual epochs were then subjected to baseline correction by removing the mean amplitude of the baseline from −300 to −100 ms prior to the onset of the stimulus from each trial. The choice of this baseline time window allowed us to capture pre-stimulus changes in the AEPs up to 100 ms prior to the onset of pitch shifts. The individual trials were then averaged to calculate the AEP for each recording contact. Figure 1a shows an example of the calculated AEP in response to pitch-shift stimuli during speaking for a single contact over the superior temporal gyrus in a subject implanted on the right-hemisphere (R186).

As seen in the example AEP waveform (Fig. 1a), responsive STG sites typically had polyphasic morphologies including large negative deflections occurring 100–250 ms after PSS onset. Therefore to perform statistical comparisons, we first compared the mean voltage of the largest negative peak of AEPs within the 100–250 ms window against the mean of the pre-stimulus waveform from −300 to −100 ms (baseline) using a paired t-test for speaking and playback conditions separately. T-tests were corrected for multiple comparisons accounting for the total number of electrode contacts using the false discovery rate (FDR) method with q = 0.01 [29]. Those contacts with AEP negativities significantly different from baseline in either speaking or playback condition (or both) were then submitted to a second FDR-corrected (q = 0.01) paired t-test to further investigate statistical difference between responses to pitch-shift stimuli during speaking compared to playback.

The spectral power of the recorded brain activity was similarly calculated on an individual trial basis using the “spectrogram” function in MATLAB with a 256 ms Hamming window and 1 ms time steps for frequencies from 0 to 300 Hz. Event-related band power (ERBP) was extracted separately for each frequency band by calculating the log transform of normalized power (relative to baseline from −300 to −100 ms) across the time-series within event-related epochs ranging from −300 to 1000 ms from stimulus onset. The log transformation function was used to ensure that the data are normally distributed for statistical analysis. The single-trial spectral power time series were then averaged over the total number of trials for speaking and playback conditions separately and results were represented as a time-frequency plot of power for each individual contact. Figure 1a shows an example of this plot for a single contact over the STG in response to pitch-shift stimuli during speaking in a subject with right side implantation (R186). Averaged spectral power traces were then computed for seven different frequency bands: delta: 0–4 Hz, theta: 4–8 Hz, alpha: 8–12 Hz, beta: 12–30 Hz, gamma: 30–70 Hz, high gamma: 70–150 Hz and very high gamma: 150–300 Hz.

The difference between power responses to pitch-shift stimuli during speaking vs. playback was analyzed for each of these seven frequency bands separately by first dividing the post-stimulus averaged power traces into 5 ms time bins and then statistically comparing the mean of each bin with the mean of baseline (−300 to −100 ms) for the same frequency band using a paired t-test. T-tests were FDR-corrected (q = 0.01) accounting for the total number of time bins and electrode contacts. For each frequency band, significant power changes (relative to baseline) for speaking and playback were determined if the mean power was significantly different from that of the baseline for at least 6 consecutive time bins (30 ms) within the post-stimulus time window. In those contacts where such significant changes in power were identified in one of the speaking or playback (or both) conditions, a second FDR-corrected paired t-test (q = 0.01, accounting for the number of contacts) was performed to assess differences between power responses to speaking vs. playback. Contacts meeting these criteria were considered to be modulated by condition in response to pitch shifts for a specific power band if the difference between the mean of power during speaking and playback was at least significant for six consecutive time bins (30 ms). Such modulation could be seen to be either an increase or a decrease in power as a result of speaking compared with playback condition.

The relative amount of change in the AEP and high gamma power responses during speaking compared with playback was quantitatively measured by calculating the modulation index (MI) in all contacts for which the AEP and high gamma responses were significantly different during speaking compared with playback. For the AEPs, the MI was defined as (AEP_Speaking_−AEP_Playback_)/(|AEP_Speaking_|+|AEP_Playback_|) in which AEP_Speaking_ and AEP_Playback_ were the peak amplitude of the AEP responses during speaking and playback conditions respectively. Similarly, the MI for the high gamma responses was defined as (HG_Speaking_−HG_Playback_)/(|HG_Speaking_|+|HG_Playback_|) in which HG_Speaking_ and HG_Playback_ were the peak magnitude of the high gamma power responses during speaking and playback. Results of this analysis are separately shown for the AEP and high gamma responses by plotting color-coded circles over the contacts that showed significant AEP or high gamma modulation. In the figures, a positive MI (graded red circles) indicates a significant enhancement of responses during speaking compared with playback and graded colors show the degree of speaking-induced enhancement with MI = +1 for maximum enhancement. Accordingly, a negative MI (graded blue circles) indicates a significant speaking-induced attenuation of responses and value −1 implies maximum attenuation.

### Voice Response Analysis

Vocal responses to downward (−100 cents) pitch-shift stimuli were calculated by extracting the pitch frequency of the recorded voice signals in Praat [30] using an autocorrelation method and then exporting them to MATLAB (Mathworks, Inc.) for further processing. The extracted pitch frequencies were then converted from Hertz to Cents scale using the formula: Cents = 1200×Log2(F2/F1) in which F1 is the mean of the pre-stimulus (−250 to 0 ms) pitch frequency and F2 is the post-stimulus pitch frequency for each trial. The extracted pitch contours were then segmented into epochs ranging from −250 ms pre- and +1000 ms post-stimulus time intervals and separately averaged for each subject. The post-stimulus time window was divided into 5 ms bins and the magnitude of the voice F0 response within each bin was statistically compared with the mean of pre-stimulus F0 (−250 to 0 ms) using FDR-corrected (q = 0.05) paired t-tests accounting for the total number of time bins. The latency of the vocal response onset was extracted by finding the time point for which the post-stimulus F0 remained significantly above the pre-stimulus F0 (baseline) for 3 consecutive time bins (15 ms). Accordingly, vocal response offset was extracted by finding the point where voice F0 returned back to the baseline and was not significantly different from the baseline for 15 ms. In addition, the latency and magnitude of the vocal response peaks were extracted by finding the global maximum of the post-stimulus F0 that was significantly larger than the mean of the pre-stimulus F0.

## Results

We observed a consistent response pattern on STG auditory cortex in response to PSS in our 10 subjects. Figure 1b illustrates the time-frequency ERBP plots (0–300 Hz) to pitch-shift stimulus for speaking and playback conditions in all 96 electrode contacts for an exemplar subject with right hemisphere coverage (R186). The AEP responses (solid black traces) for each contact are also overlaid on these plots. The gray solid vertical lines in each plot marks the onset of PSS (time: 0 ms) and event-related epochs are plotted for −300 to 1000 ms relative to the PSS onset. As can be seen, PSS elicited a focal cluster of ERBP and AEP responses in a limited area over the STG in the vicinity of the lateral termination of the transverse temporal sulcus (TTS) for both speaking and playback conditions. Further analysis showed that the significant increases in power (relative to baseline) were most prominent in the high gamma (70–150 Hz) range, and from the anatomical standpoint, these responses were closely co-localized with areas of significant AEP responses during both speaking and playback. Close inspection within this response cluster showed that despite the overlap between areas of high gamma and AEP responses, significant increase in either high gamma or AEP did not always precisely co-localize. Also, some contacts within this focal response cluster showed varying degrees of vocalization-induced modulation of PSS responses. Figure 1c demonstrates examples of three individual contacts on STG that exhibit differing effects. As can be seen, in contacts 68, 69 and 76 (marked with red, blue and brown circles respectively), significant high gamma and AEP responses to PSS are elicited for both speaking and playback conditions. However, in contact 68 (red), both high gamma and AEP amplitudes are significantly enhanced during speaking compared with playback whereas for contact 69 (blue), the significant amplitude enhancement is only observed for the AEP. Finally, at a site just 5 mm anterior at contact 76 (brown), no such statistical differences are observed between the ERBP and AEP responses during speaking compared to playback and the responses have very similar morphologies indicating no speech-related modulation.

**Figure 2:**
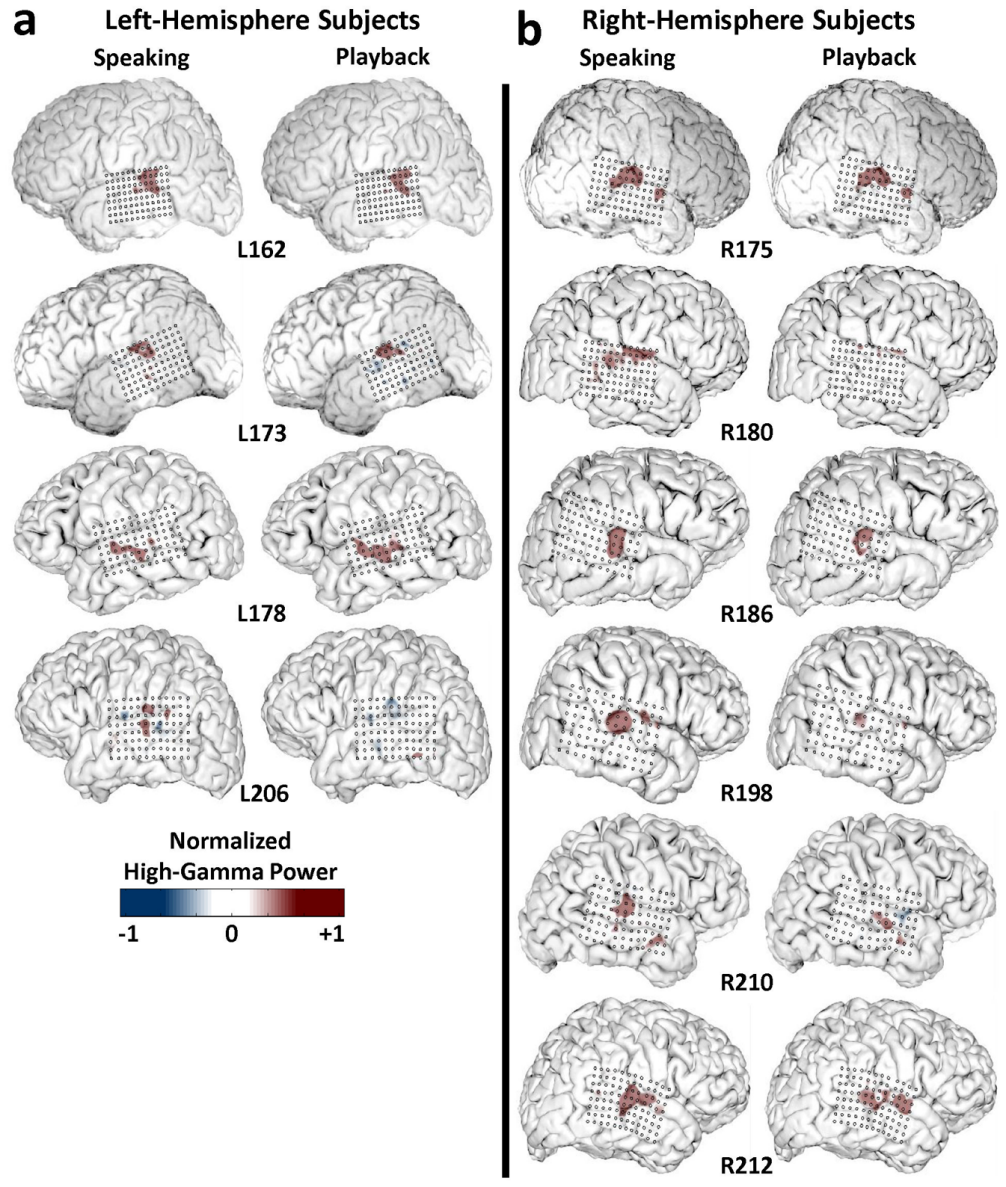
Left and Right STG High Gamma Response Patterns to PSS. Topographical maps for each subject demonstrating the consistent focus of high gamma (70–150 Hz) power increases on the posterior portion of STG in both left (a) and right (b) hemisphere subjects. Power responses are depicted as normalized values (+/−1) based on the contact for each subject demonstrating maximal high gamma power amplitude in a 0–500 ms post-pitch shift stimulus time window. In each sub-panel, the distribution maps are shown for responses to pitch-shift stimuli during speaking (left) and playback (right) conditions, separately.

Like this exemplar subject (R186), the other 9 subjects also demonstrated this response pattern of a focal subregion of posterior STG showing AEP and high gamma responses to PSS. In all 10 cases the responses clustered immediately adjacent to the TTS in recordings from both left and right hemisphere subjects. This anatomic consistency is illustrated by plotting the topographical distribution of the normalized mean of the high gamma power within a 0–500 ms post-stimulus time window in figure 2. For each subject, normalization is performed through dividing the mean of the high gamma power (0–500 ms) in each contact by the largest high gamma power in the whole grid (96 contacts). As can be seen for the left (Figure 2a) and right (Figure 2b) STG subjects, both speaking and playback PSS responses are observed as increases in high gamma power compared to the pre-PSS baseline amplitude in discrete STG cortical areas. There is some variability noted in the rostral-caudal extent of STG activation between subjects, but the center of high gamma power activation is observed at the level of the TTS.

The focal nature of the high gamma responses observed on STG is reflected in the fact that of the total number of 960 recording contacts (96 contacts in 10 subjects), significant high gamma responses (relative to baseline) were elicited in only 180 (19%) and 112 (12%) individual contacts during speaking and playback conditions respectively. A total number of 86 (9%) contacts had significant high gamma responses during both speaking and playback conditions. A FDR-corrected (q = 0.01) paired t-test showed that the onset latencies of high gamma responses were significantly shorter during speaking (mean: 150.7 ms, std: 5.9 ms) compared with playback (mean: 176.1 ms, std: 7.5 ms) condition (p<0.001). For the majority of these contacts, the high gamma responses remained significantly above baseline for about 200–300 ms and returned to their baseline level in about 500 ms after the onset of the stimulus.

**Figure 3:**
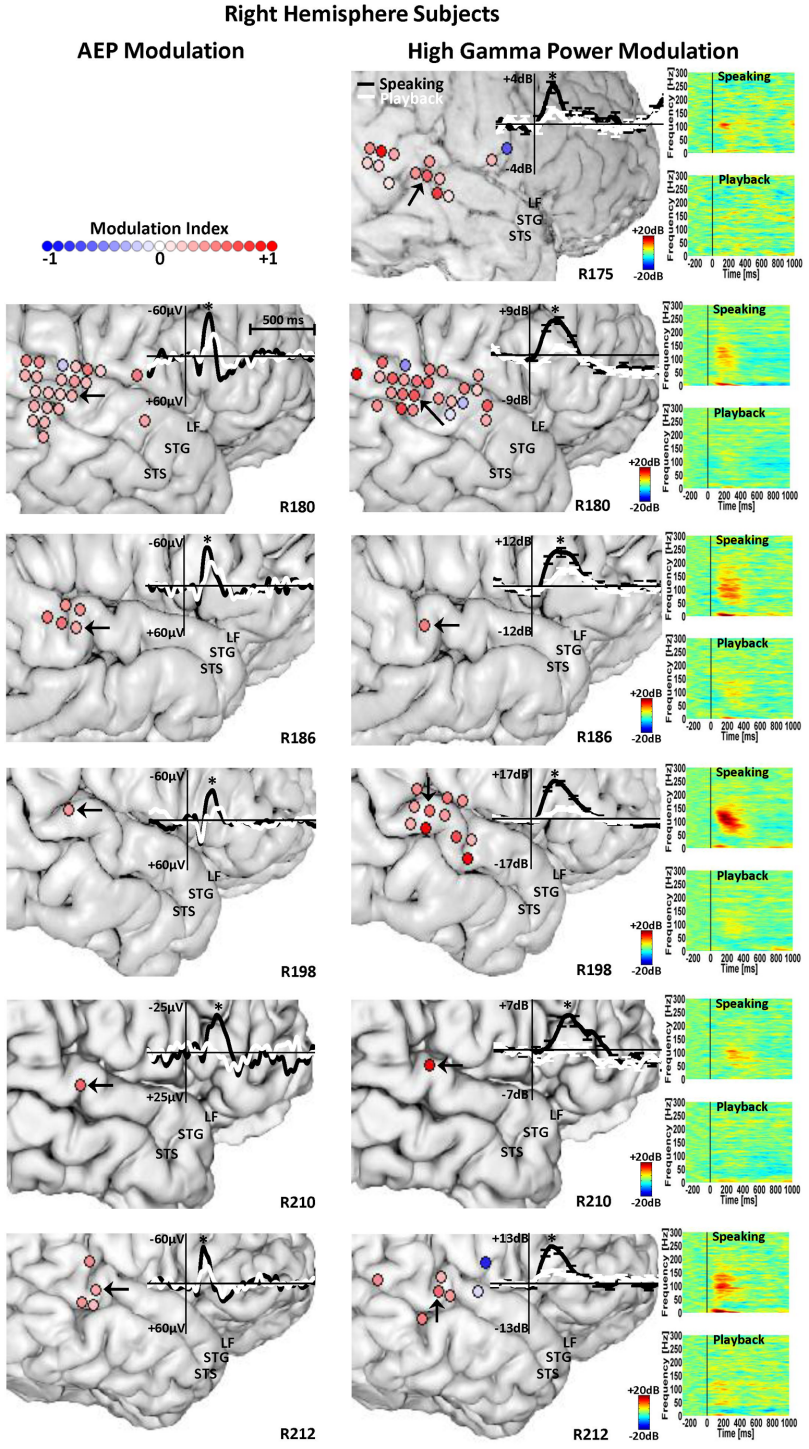
Right STG AEP and HGP Modulation. Anatomical location of contacts in subjects with right-hemisphere temporal lobe implantation that show significant AEP (left panel) and high gamma (70–150 Hz) power modulation (right panel) in response to PSS during speaking compared with playback. The graded color-coded circles at each contact site correspond to the degree of the speaking-induced enhancement (red circles) or attenuation (blue circles) of the AEP and high gamma responses, measured by the modulation index. In the left panel, the overlaid AEP traces are shown for a STG contact (black arrow) with significant speaking-induced AEP enhancement for each subject. In the right panel, the overlaid high gamma power traces along with time-frequency plots of the spectral power are shown for a STG contact (black arrow) with significant high gamma power enhancement during speaking compared with playback.

**Figure 4:**
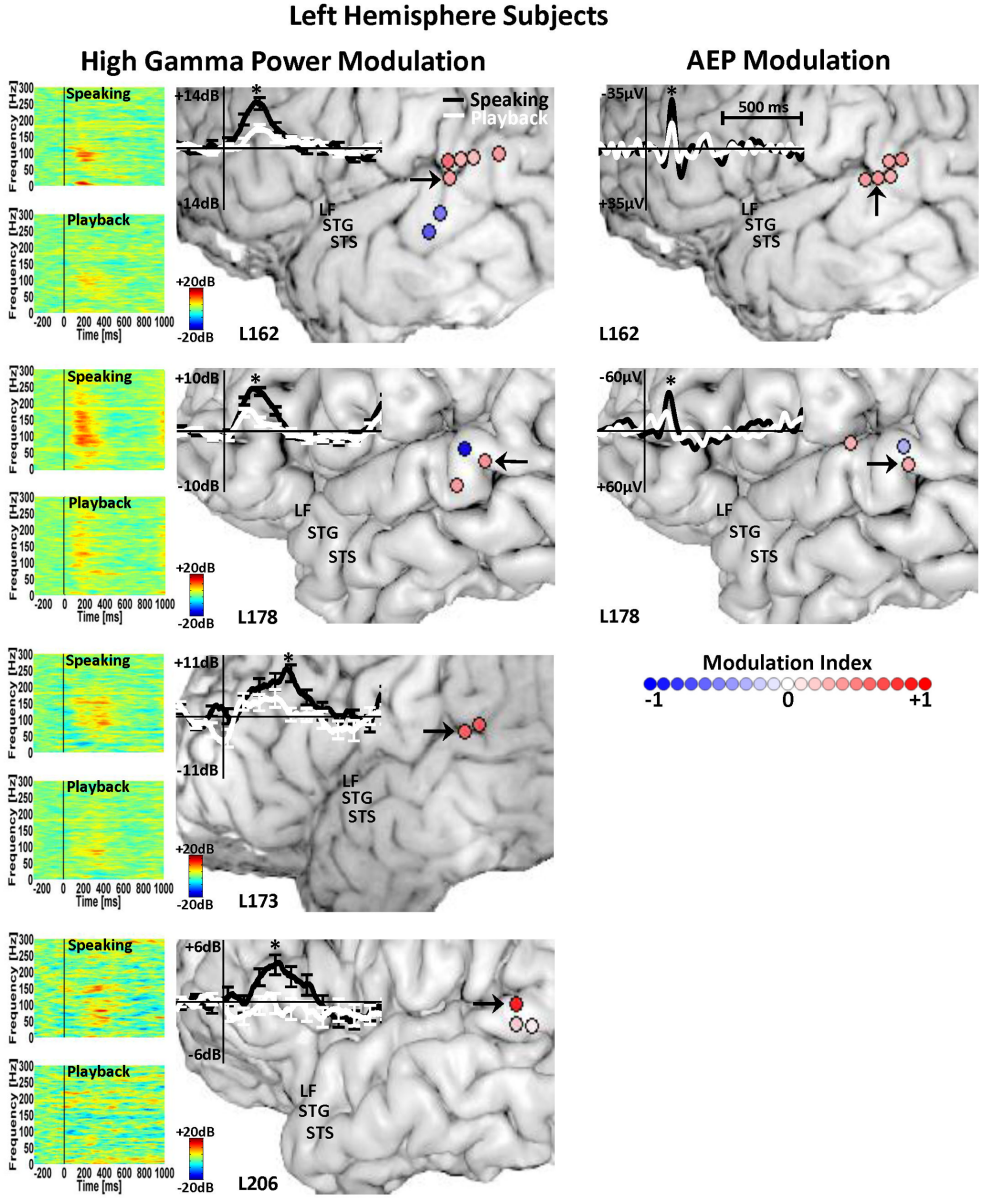
Left STG AEP and HGP Modulation. Anatomical location of contacts in subjects with left-hemisphere temporal lobe implantation that show significant AEP (right panel) and high gamma (70–150 Hz) power modulation (left panel) in response to PSS during speaking compared with playback. The graded color-coded circles at each contact site correspond to the degree of the speaking-induced enhancement (red circles) or attenuation (blue circles) of the AEP and high gamma responses, measured by the modulation index. In the right panel, the overlaid AEP traces are shown for a STG contact (black arrow) with significant speaking-induced AEP enhancement for each subject. In the left panel, the overlaid high gamma power traces along with time-frequency plots of the spectral power are shown for a STG contact (black arrow) with significant high gamma power enhancement during speaking compared with playback.

Modulation of STG responses to PSS by vocal production is evident through statistical comparisons of high gamma responses between speaking and playback conditions (Figures 3 and 4). Results from every subject are depicted with full spectrogram plots for each condition along with overlaid high gamma contours for speaking and playback for an example STG contact (marked with a black arrow). These example contacts all showed *enhancement* of high gamma power during speaking compared to playback. Conversely, there were some sites on STG that showed *attenuation* of high gamma power during speaking compared to playback. A total number of 74 contacts exhibited speaking-induced modulation and the vast majority (65 contacts, 88%) showed enhancement in high gamma responses as opposed to attenuation (9 contacts, 12%). Sites demonstrating these different response types were in close (<15 mm) anatomic proximity. The calculation of the MI showed variable degrees of speaking-induced modulation of high gamma responses to pitch-shifted voice feedback (Figures 3 and 4). The mean value of the MI for contacts showing high gamma enhancement and attenuation during speaking was 0.42 (std: 0.23) and −0.45 (std: 0.3), respectively.

Examination of the AEP responses to PSS revealed similar response patterns as that of the high gamma responses. The overall extent of activation as indicated by statistically significant differences of AEP amplitude compared to baseline was similar to high gamma responses. Namely, across all subjects, 220 (23%) and 114 (12%) contacts significantly responded to pitch-shift stimuli during speaking and playback respectively. Within these contacts, 95 (10%) contacts had significant AEP responses during both speaking and playback conditions. A FDR-corrected (q = 0.01) paired t-test did not show a significant difference between AEP latencies during speaking (mean: 182.3 ms, std: 5.3 ms) compared with playback (mean: 179 ms, std: 5.9 ms) condition. Statistical comparison between AEP amplitudes during speaking and playback revealed significant differences in 41 (4%) contacts, representing response modulation during speaking (Figures 3 and 4). Of the sites showing response modulation, 39 (95%) contacts had AEPs that were *enhanced* (larger amplitude) during speaking compared with playback and only 2 (5%) contacts showed AEP *attenuation* during speaking. The mean value of the MI for contacts showing AEP enhancement and attenuation during speaking was 0.36 (std: 0.09) and −0.27 (std: 0.08), respectively.

For the contacts that showed a significant modulation of both the AEP and high gamma responses (total of 21 contacts), results of the analysis showed the AEP and high gamma MIs were not correlated (Pearson’r Correlation = 0.08, p = 0.745). This indicates that the degree of speaking-induced enhancement or attenuation in the high gamma power responses was not correlated with the degree of AEP modulation during speaking.

Even though AEPs were elicited in response to PSS in all ten subjects, significant differences across conditions were identified in 5 of 6 subjects with right hemisphere (Figure 3) and 2 of 4 subjects with left hemisphere implants (Figure 4). This means that the AEPs were not significantly modulated by speaking in three subjects (L173, R175 and L206), although all 3 subjects had modulation of high gamma responses. In Figures 3 and 4, the overlaid AEPs during speaking and playback are also plotted for an example STG contact from each subject which demonstrated response enhancement during the speaking condition. As can be seen in these figures, for the majority of the subjects, speaking-induced enhancement of the AEPs follows a consistent anatomical distribution pattern (like that of high gamma responses) and occurs over a limited area of STG near the TTS. One subject (R180) had AEP response modulation extending over the superior temporal sulcus into the middle temporal gyrus as well as a single contact on the posterior inferior frontal gyrus (Figure 3). Similar to these AEP responses, in some subjects (e.g. R180, R198 and R212), the anatomical distribution of high gamma modulation extended beyond the STG and included areas such as middle temporal and inferior frontal cortices (Figures 3 and 4).

In the total number of 10 subjects, vocal responses could not be measured in one subject (L162) due to the fact that during the recording session, the voice and feedback signals were mixed and recorded together on one channel and therefore voice F0 contour could not be separately extracted for this subject. For subject R210, none of the post-stimulus time bins were significantly different from the mean of the pre-stimulus F0 (baseline). For the 8 remaining subjects, analysis of the vocal responses showed that each of these 8 subjects significantly compensated (q<.05) in response to downward (−100 cents) feedback pitch perturbation by raising their vocal pitch output in the upward direction. All subjects except for R175 returned their voice F0 to the baseline after compensating for downward pitch perturbation in their voice auditory feedback. The measures of the vocal response onset, offset and peak latencies along with the peak magnitude for all subjects are summarized in Table 1. Group-wise analysis of vocal response peak magnitude confirmed a significant compensatory effect (p = .04, paired t-test).

**Table 1:** Measures of the compensatory vocal responses to downward (−100 cents) pitch-shifts in voice auditory feedback.

Subject	Onset Latency (ms)	Offset Latency (ms)	Peak Latency (ms)	Peak Magnitude (cents)
L173	220	695	400	20.6
L178	285	765	405	6.3
L206	185	290	235	17.6
R175	175	–	330	12.3
R180	180	245	205	9.5
R186	115	650	240	43.9
R198	170	410	275	12.3
R212	145	815	295	32.4
**Mean (std)**	**184 (51)**	**553 (234)**	**298 (75)**	**19.4 (12.8)**

## Discussion

The present study provides evidence for speaking-induced modulation of auditory cortical responses to unexpected perturbations in voice pitch feedback. We found that the pitch-shift stimuli elicit AEP responses within non-primary areas of human auditory cortex (mainly posterolateral STG) that are enhanced during active vocal production of a vowel sound as compared to passive listening to playback of the same self-produced vocalizations. ERBP responses were found to be most prominently elicited within high gamma frequency range (70–150 Hz) and similar to the AEPs, these responses were predominantly *enhanced* during speaking compared with playback condition. In addition, a number of contacts were identified within the STG that although they were clearly responsive to the acoustic tasks, they showed no evidence of speaking-induced modulation of AEP or high gamma responses. Moreover, our data show that even though the AEP and high gamma responses were similarly localized within circumscribed regions of the STG near the TTS, the anatomical pattern of speaking-induced modulation did not fully overlap for the AEP and high gamma responses. Calculation of the modulation index (MI) measure indicated variable degrees of speaking-induced AEP and high gamma modulation within the STG for each individual subject and we did not observe a consistent pattern of spatial distribution for the extent of modulation across a group of 10 subjects. As can be seen in figures 3 and 4, a large number of contacts can be identified that showed a significant speaking-induced modulation of the AEPs but not the high gamma responses and vice versa. However, a number of contacts showed simultaneous modulation of both the AEP and high gamma responses during speaking but the results of the analysis for these contacts did not show a significant correlation between the MIs for these two types of responses.

These findings suggest that encoding pitch error in voice auditory feedback for vocal motor control is mediated by complex neural processes in distinct cortical networks within the non-primary human auditory cortex. These neural processes are reflected through the activity of neural systems that generate both low and high-frequency oscillations. The low-frequency components of these oscillations emerge as neural activities that are evoked by pitch perturbations and contribute to generation of high-amplitude phase-locked AEP responses as well as increased induced ERBPs at frequencies below 20 Hz. The low frequency AEP components are thought to be generated by more global neural circuits that orchestrate synchronized activity between different regions for performing specific functions [31]. These phase-locked potentials are proposed to be associated with post-synaptic dendritic membrane potentials that are locally generated as a result of receiving synaptic input from subcortical structures for bottom-up representation of stimulus-specific features or from other cortical regions for top-down cortico-cortical interactions [32]. In contrast, the high-frequency (e.g. high gamma: 70–150 Hz) oscillations have been suggested to co-vary and be tightly correlated with spiking activity [33], and therefore, reflect distinctly different functional mechanisms as compared with low-frequency generators. The high gamma oscillations are comprised of both induced (non-phase-locked) and evoked (phase-locked) components [34] and are associated with synchronized neuronal firing in spatially segregated, but functionally related cortical regions. Thus, the high frequency oscillations can be considered to represent mechanisms by which local neuronal assemblies are bound together to process higher-order stimulus properties.

In this context, one possible interpretation of the AEP and high gamma responses to pitch-shifted feedback in the present study is that they may reflect mechanisms of stimulus feature representation as well as sensory-motor interactions that facilitate feedback-based monitoring of voice for vocal pitch control. It is possible that the evoked AEPs in the present study may arise from changes in post-synaptic membrane potentials of neuronal assemblies in a focal subregion of the STG that receive synaptic input from lower-order subcortical and cortical auditory pathways as well as other non-auditory cortical areas (e.g. prefrontal, motor, parietal etc.). Based upon our data, it can be suggested that the cortical networks within those areas that show a significant AEP response without speaking-induced modulation of AEP activity may only involve neural processes that encode the features of the pitch-shift stimulus in the auditory feedback. However, those regions that exhibit a significant modulation of AEP responses during speaking may include neural circuits that receive synaptic input from cortical vocal motor areas and may serve as a neural substrate for comparison between sensory (i.e. auditory) and internally-predicted feedback (e.g. efference copies) for detecting pitch perturbations. Therefore, it can be suggested that access to internal predictions can selectively enhance neural sensitivity to feedback perturbations and consequently lead to the elevation of post-synaptic membrane potentials within specific cortical auditory networks.

With relevance to this discussion, the high gamma responses to feedback pitch perturbations can be interpreted as an indication of increased spiking activities as a result of auditory representation and perception of pitch shifts in voice feedback as well as the output of pitch error detection mechanisms that drive the compensatory vocal responses through long-range synaptic projections from the auditory to vocal motor cortex. Our data suggest that different cortical auditory networks subserve these two distinct functional mechanisms as it is shown that the high gamma responses are modulated during speaking compared with playback only in a focal sub-region of the STG in the vicinity of the transverse temporal sulcus. Speaking-induced modulation of high-gamma activity has also been reported in a recent study showing that the enhanced responses are mainly distributed in the posterior portion of the STG along with ventral pre-motor areas and high-gamma enhancement in these areas is correlated with the peak of vocal compensation magnitude during speaking [35]. Based on these findings, we propose that the posterior sub-region of the STG may be an important anatomical landmark for auditory feedback processing and may be part of specific neural circuitries for audio-vocal interactions during vocal pitch control. In addition, results of our present study indicating the absence of a complete overlap between the spatial distribution of AEP and high gamma response modulation during speaking in adjacent contacts (only 5 mm apart) suggests that the functional properties of networks generating these two types of responses can abruptly change between lateral anatomical areas within the columnar structure of non-primary human auditory cortex.

Early evidence for motor-induced modulation of auditory cortical processing is provided by studies showing that induced vocalizations via electrical stimulation of the central gray caused reductions of spontaneous firing rates of neurons within auditory cortex of squirrel monkeys [12], [36]. This effect was confirmed by studies during voluntary vocalizations in marmosets [10], [11] and humans [14]–[16], [18], [19]. The forward model theory [1], [37] posits that motor-induced suppression of auditory activity results from neural processes that compare actual voice feedback with an internal representation of the predicted vocal output. It has been proposed that these internal predictions may be partially generated by efference copy mechanisms [2]–[4] that use a neural representation of vocal motor commands to predict the sensory (e.g. auditory) consequences of self-produced vocalizations. This notion was supported by showing that motor-induced suppression is reduced when internal predictions are violated by external perturbations in voice pitch auditory feedback [13], [17]. The ability to recognize disparities between predicted and actual voice feedback is proposed to be one strategy by which the brain identifies the source of incoming sounds and distinguishes self-voices from those generated by other speakers [20].

Despite the generally suppressive nature of motor-driven modulation of auditory cortical activity, studies have suggested that efference projections play a key role in increasing neural sensitivity for detecting unexpected perturbations in voice auditory feedback. Single unit recordings in marmosets’ auditory cortex show that the majority of suppressed neurons during vocalization increased their firing rates in response to pitch-shifted feedback [22]. In humans it has been demonstrated that the amplitudes of scalp ERP responses to pitch-shifted voice feedback are larger during vocalization compared with listening, and this vocalization-induced enhancement becomes smaller as the magnitude of the pitch-shifts (error) is increased [21]. Findings of the present study support these reports since the direct recording of ECoG from the lateral surface of human auditory cortex exhibit AEP and high gamma power enhancement in response to pitch-shifts during speaking compared with playback. Our results suggest that neural circuits involved in monitoring voice auditory feedback are located within a limited region of lateral STG in the vicinity of TTS on both left and right hemispheres. Hemispheric differences in pitch processing have been reported in prior studies postulating that the right auditory cortex is specialized for processing spectral cues whereas the left is mainly involved in tracking temporal dynamics of auditory stimuli [38]–[42]. Though our results showed auditory cortical activation in the left and right sides, we could not investigate laterality effects in individual subjects because unilateral grid implants were used in all cases.

The patterns of STG activation observed in the present study are consistent with data from functional imaging. For example, bilateral STG activation was demonstrated in response to normal and pitch-shifted voice auditory feedback during vowel phonations [43]. Similar effects within bilateral STG, right prefrontal and peri-Rolandic cortical regions were reported when the first formant frequency was shifted in auditory feedback during production of monosyllabic words [44]. It has been shown that voluntary changes in voice F0 in response to long-duration (500 ms) pitch perturbation is associated with activation in STG along with putamen, insula, anterior cingulate cortex and pre-motor cortex in singers and non-singers [45], [46]. A model of the neural circuitry of the sensory-motor mechanisms of speech production and control has proposed that STG responses to auditory feedback perturbations are related to the activity of “error cells” that detect feedback changes by comparing incoming sensory feedback with an internal representation of the speech sound map provided by feedforward mechanisms of the vocal motor system [47].

The present findings can be discussed in the context of proposed audio-vocal integration models of speech production and control [48], [49]. Based on these models, vocal control involves interactions between pre-motor, motor, auditory, parietal and inferior frontal cortices. In a dual auditory processing framework, the streams are identified as distinct postero-dorsal (PD) and antero-ventral (AV) pathways [50]. The AV pathway consists of projections from the anterior belt regions of auditory cortex to ventro-lateral prefrontal cortex (VLPFC), transforming auditory representations to motor commands that drive pre-motor and motor cortex for speech production. The projections from VLPFC to inferior parietal cortex (IPC) and posterior superior temporal gyrus are proposed to transmit feed-forward signals that carry an internal representation of sensory (i.e. auditory) feedback associated with self-produced speech in the form of efference copies. The PD pathway consists of projections from posterior belt regions of auditory cortex interfacing with pre-motor cortex via direct synaptic projections and indirect projections through the IPC, providing auditory feedback information that is compared with internally predicted motor commands. If the general structure of this model holds true, it suggests STG and IPC as candidate areas where neural representations of actual and predicted voice interact to detect and correct for feedback errors. The present study provides evidence of the involvement of higher order auditory cortex in this postulated efference copy system, in that the act of vocalization enhances neural sensitivity to feedback pitch perturbations within circumscribed regions of lateral STG. The observation that vocalization-induced modulation of speech sound processing was restricted to certain regions of auditory cortex suggests the presence of selective functional connections linking specific motor and sensory brain regions. We are pursuing additional studies using a variety of experimental techniques to identify and characterize these putative pathways.

Even though direct evidence for the presence of a motor-related activity in the sensory (e.g. auditory) system has not previously been established, a large number of studies have provided supporting evidence for the existence of an efference copy mechanisms that can modulate neural processing of sensory feedback in different sensory (e.g. visual, auditory, somatosensory) modalities [1], [10], [15], [17], [20]–[22], [37], [51], [52]. However, one important consideration is that in addition to the proposed top-down effects of the efference system, other factors such as increased attention to voice feedback during speaking may have also contributed to the modulation of the AEP and high gamma responses for speaking vs. playback conditions in the present study. We believe, although the attentional effects may have partially contributed to the difference in neural responses during speaking vs. playback, they cannot fully explain the observed modulation of AEP and high gamma responses in the present study. This is mainly because during speaking we observed non-uniform degrees of speaking-induced modulation of sensory responses. Calculation of the MI measure for all contacts showed that the AEP and high gamma modulations ranged from highly-enhanced to highly-suppressed at some contacts in different parts of the STG. This effect is contrary to the expected effect of attention as it would enhance neural responses during the active task (speaking) compared with listening. In addition, previous studies of vocal production have shown a completely opposite effect as the neural responses at majority of contacts were suppressed during speaking. These data suggest that the modulation of sensory responses during speaking cannot be merely explained by higher attentional loads during the active task of speaking and may be attributed to activations of other mechanisms (e.g. efference copies) that may enhance neural sensitivity to auditory feedback perturbation during vocal production and control.

Lastly, the analysis of the behavioral data showed consistent results in terms of the nature of vocal responses to pitch-shifts in the auditory feedback. A large sub-group of the tested subjects (8 out of 10) exhibited an upward (compensatory) change in their voice pitch in response to downward stimuli. However, we observed a large amount of variability in response properties of the vocal behavior (see Table 1) and it was difficult to determine how this variability may relate to the variability in the recorded neural data. For example, subject R210 showed strong high gamma modulation during speaking in one contact over the STG (MI: 0.89) without showing a significant vocal response to feedback pitch perturbation. In contrast, the high gamma modulation in subject R186 was moderate (MI: 0.42) but this subject showed the largest vocal compensation magnitude (43.9 cents) in the tested group of subjects This inconsistency between the measures of vocal and neural responses may arise because vocal responses are likely to be more variable across different subjects due to the fact that they can be affected by neural mechanisms in different areas (e.g. cortical, cerebellum, limbic system, brainstem) and varying biomechanical and muscular parameters of the laryngeal and respiratory systems [53]–[57]. Future studies would be needed to investigate the neural correlates of auditory feedback processing with respect to the induced changes in vocal behavior using a variety of experimental procedures.
